# Decision-making on therapeutic futility in Mexican adolescents with cancer: a qualitative study

**DOI:** 10.1186/s12910-017-0231-8

**Published:** 2017-12-11

**Authors:** Carlo Egysto Cicero-Oneto, Edith Valdez-Martinez, Miguel Bedolla

**Affiliations:** 1 Haemato-Oncology Department Hospital Infantil de Mexico “Federico Gómez”, National Health Institute of the Secretariat of Health, Mexico City, Mexico; 2 Health Research Council of the Mexican Institute of Social Security, Centro Medico Nacional Siglo XXI, Av. Cuauhtemoc #330. Col. Doctores, C.P. 06720 Ciudad de Mexico, Mexico City, Mexico; 3 Policy Studies Centre of the College of Public Policy, University of Texas in San Antonio, San Antonio Texas, USA

**Keywords:** Cancer, Palliative care, Adolescents, Parents, Physicians, End-of-life, Decision-making, Mexico

## Abstract

**Background:**

The world literature shows that empirical research regarding the process of decision-making when cancer in adolescents is no longer curable has been conducted in High-income, English speaking countries. The objective of the current study was to explore in-depth and to explain the decision-making process from the perspective of Mexican oncologists, parents, and affected adolescents and to identify the ethical principles that guide such decision-making.

**Methods:**

Purposive, qualitative design based on individual, fact-to-face, semi-structured, in-depth interviews. The participants were thirteen paediatric oncologists, 13 parents or primary carers, and six adolescents with incurable cancer. The participants were recruited from the paediatric oncology services of three national tertiary-care medical centres in Mexico City.

**Results:**

The oncologists stated that they broach the subject of palliative management when they have determined that curative treatment has failed. Respect for autonomy was understood as the assent of the parent/adolescent to what the oncologist determined to be in the best interest of the adolescent. The oncologists thought that the adolescent should be involved in the decision-making. They also identified the ability to count on a palliative care clinic or service as an urgent need. For the parents, it was essential that the oncologist be truly interested in their adolescent child. The parents did not consider it necessary to inform the child about impending death. The adolescents stated that the honesty of their oncologists was important; however, several of them opted for a passive role in the decision-making process.

**Conclusion:**

The findings of this study evidence that to achieve good medical practice in low-middle income countries, like Mexico, it is urgent to begin effective implementation of palliative care, together with appropriate training and continuing education in the ethics of clinical practice.

**Electronic supplementary material:**

The online version of this article (10.1186/s12910-017-0231-8) contains supplementary material, which is available to authorized users.

## Background

A 2014 systematic review [1] of studies, published between 1988 and 2012, shows that the majority of empirical research about the decision-making process when cancer treatment in children and adolescents is no longer curative is based primarily upon interviews with bereaved parents, and to a lesser extent, on interviews with attending oncologists, and on reviews of medical records. Only one study included statements from the affected young people. All these studies were carried out in High-income, English speaking countries.

The results from these studies cannot be extrapolated to Mexico because of the characteristics of its culture, especially those relating to feelings, values, beliefs, and patterns of communication in the decision-making process during medical treatment. Primary studies in the international literature [2, 3] show how different systems of values and ethnic identities influence the preferences of the parents as to informing their children, or not, about their imminent death. For example, in contrast to white Americans, the Japanese, the Chinese, and the Dutch avoid discussing death with a child with incurable cancer.

Lonergan [4] proposes that a culture is a set of skills, feelings, values, and beliefs that are shared by people in such a way that they can collaborate in the construction of what is good for the individual, for society, and in the end, for all human beings. Mexicans have a rich culture with many traditions. They call themselves “mestizos” (mixed), because their culture was shaped by their Meso-American (indigenous peoples), European, and African ancestors [5]. Mexican culture is also strongly influenced by the dominant culture of the USA, its neighbour to the north. As a result, in medicine, illness and death tend to be viewed through the lens of biomedical explanation and Mexican culture plays a peripheral role.

Although there is no single definition of the “good death”, it could be defined as one that is free from avoidable distress and suffering in patients, carers and families; and is in accord with patients’ and families’ wishes; while also being consistent with cultural, clinical and ethical standards for this stage of life [6, 7].

The Mexican healthcare system faces two principal challenges in providing high-quality medical attention to adolescents with cancer. First, for adolescents 15–19 years old, cancer is the 4th leading cause of death after accidents, assault and battery, and injuries; thus, cancer is the primary cause of death due to illness [8]. Mortality from cancer in adolescents is greater than that for those 0–14 years old, with an estimated 56% survival from the time of diagnosis [9]. According to Mexican national statistics, 21% of adolescents with cancer are attended in public hospitals belonging to the Mexican social security system and 79% in public hospitals that provide medical attention to those that do not have social security [9]. It should be noted that the Mexican healthcare system comprises public and private components, serving ~95% and ~5% of the population, respectively. In the public sector are the institutions of social security and the institutions and programs that attend the population without social security [10]. Although many hospitals report palliative care services, most of them function solely as “pain clinics” [11, 12]. There is no reliable estimation of the nature of these services. The available data are product of personal appreciations obtained by telephonic or email surveys that provide results that do not coincide among them [11, 12]. Nonetheless, it is estimated that <5% of the hospitals provide palliative care services to children and adolescents with cancer.

The second challenge derives from the wide variation in the quality of the medical attention that the population receives within the Mexican healthcare system. In a report published by *The Economist* on for the overall Quality of Death index, Mexico had a low score (2.7 out of 10), ranking 36th among 40 countries [13]. There is a lack of statistical data about Mexican adolescents; however, it is documented that, in general, in developing countries, like Mexico, >80% of patients with cancer suffer pain before dying [14]; pain is consistently one of the most feared consequences of cancer for patients and families [14]. In December 2014, the Mexican government, with the intent of improving this situation, issued a resolution in which the Secretariat of Health declared that the provision of palliative care is obligatory [15]. This pronouncement is aligned with the World Health Assembly resolution WHA67.19 of the World Health Organization [16]. Yet, despite this resolution and its compulsory nature, both family members and healthcare professionals, particularly oncologists, have only limited knowledge of the resolution and of how to translate it into clinical practice.

A study that included the review of 63 clinical medical records of Mexican adolescents with cancer, who had died between 2011 and 2014 in three of the main tertiary-care hospitals in Mexico City, shows that, of 40 adolescents diagnosed to be in terminal phase of their disease, 16 (40%) continued to receive treatment with curative ends. Of the 51 whose place of death was known, 45 (88%) died in hospital. Of the 41 who died within 30 days of their last hospitalization, very few, three of them (7%) received palliative treatment [17].

The study here reported is both timely and relevant. The evidence generated is urgently needed in order to understand the reality of the situation of adolescents with cancer, when treatment is no longer curative. There are no published studies that address this problem in low-to-middle income countries such as Mexico. The objective of the current study was to explore in-depth, to understand, and to explain the decision-making process from the perspective of attending paediatric oncologists, parents, and adolescents with incurable cancer, and to identify the ethical principles that guide such decision-making. The results of this study will contribute to design interventions that will help clinicians to confront the moment when further curative cancer treatment is futile in order to improve the quality of death for the adolescents with cancer and to promote the well-being of their families.

### Conceptual framework

Howard’s descriptive theoretical decision analysis model [18] enables focussed exploration of the cognitive process of decision makers, and the decision-making process, to develop descriptions of how people actually make judgments and decisions. The model facilitates exploration as to how the interests, values, preferences, and goals, expressed by the principal actors, are converted into an effective reference in decision-making, when curative treatment no longer offered any benefit to the patient. Thereby, the Howard’s model permitted us to focus on the exploration of the decision-making process in order to describe how the decision-makers judge to be true the truths on which they based their decisions, rather than on the evaluation of the quality of those decisions.

## Methods

### Study design

From August 2013 to May 2015, a qualitative study was carried out which included individual, face-to-face, semi-structured, and in-depth interviews as the technique for generating information.

### Study population

Thirteen paediatric oncologists who worked the morning shift and were tenured at one of the three medical centres (see next section) participated in this study along with 13 parents of 13 adolescents with cancer. Seven of these adolescents had already died; for the remaining six, who were alive at the time of the study, their parents had been already informed of the therapeutic futility. Also participating were six adolescents; four of them were the children of this group of parents. Two parents refused to allow their children to participate in the research, stating that their children were not aware of their terminal prognosis, and that they desired to protect their children from potential information that might be harmful to them. Based on the evolution of the analysis of the information generated, it was necessary to recruit two more adolescents, whose cancer, according to their attending oncologists, was incurable or in terminal phase. Purposive sampling was used [19], with the sample size defined by theoretical saturation, according to the criteria of Sandelowski [20]. In brief, following those criteria, the sampling was stopped when the data became repetitive or redundant and new analyses only confirmed what had already been established.

### Participating hospitals

The 32 participating subjects were recruited from the paediatric oncology services of three tertiary-care medical centres located in Mexico City: one (Hospital Infantil de Mexico) belongs to the Secretariat of Health and two (General Hospital of the Medical Centre “La Raza” and Paediatric Hospital of the Medical Centre “20 de noviembre”) belong to the social security system of Mexico. These hospitals were selected because they are among the principal national referral centres, providing medical care to patients from various parts of Mexico. They have the accreditation, infrastructure, and resources necessary to provide medical attention to children and adolescents with cancer. They are also pillars of medical research in Mexico [10].

### Terminology

Here, the term “parents” is used to refer to the group of parents or primary carers who participated in the study: eight mothers, three fathers, one sister, and one grandmother (i.e., the person in charge of the care of the adolescent). The term “therapeutic futility” is used when the attending oncologist determined that the goal of curative therapy was unattainable and no longer indicated for the patient.

### Pre-interview activities

The research ethics committee of each participating hospital approved this study. Informed consent was obtained from all participants, prior to their interview. For the group of adolescents interviewed, informed consent was first obtained from the parents and then informed assent from each of them. Anonymity of the information was guaranteed by tagging the data in a manner that could not lead back to the informant. The interviewers were one male and two female psychologists (AJ, GQ, GP): one is a specialist in clinical interventions for children and adolescents and two are psychologist-oncologists for children and adolescents. They were trained in the technique of semi-structured, in-depth interviewing by (AZ) a skilled social scientist. When it was corroborated (by means of pilot tests) that they had mastered the technique for generating data, the field study was begun. To avoid possible psychologist/interviewer or physician/interviewer role conflict, interviewers were never in charge of the psychological care or medical care of those interviewed.

### Procedure

An interview topic guide was developed (“Additional file 1”), based on theoretical knowledge and group discussions with the research team. This guide underwent adaptations throughout the study, as a function of the analysis of the information being generated and new threads of questioning being identified. The topic guide included issues related to values, beliefs, preferences, interests, and ideas associated with medical care, treatment, and decision-making for adolescents with incurable cancer. Further information, such as demographic data, was also collected during the interviews. Because these interviews could raise unanticipated emotional issues, in cases of distress, interactions were guided by respondents’ emotional needs.

The individual, face-to-face interviews were conducted in private (i.e.*,* a researcher together with a psychologist and an interviewee, or two psychologists and an interviewee), at a site selected by the interviewee. For the parent group, only one parent or primary carer per family was interviewed. The first five participating oncologists had to be interviewed twice in order to clarify the information that they generated; hence, there are 32 interviews, but 37 primary documents. The interviews lasted a median of 43 min (range: 11–134 min). The interviews were audio recorded and an experienced medical transcriber (AL) transcribed all of the recordings verbatim. The researchers independently reviewed each one of the recordings with their own respective transcriptions in order to corroborate the correct emphasis of each one of the arguments.

### Analysis

The entire data/narratives set were analyzed by the method of thematic analysis [21]. Themes or patterns within data/narratives set were initially identified in an inductive way. Accordingly, the analysis involved constant, interactive, and reflexive revisions of each one of narratives, independently completed, by the two researchers (CC and EV); any discrepancy was resolved by consensus. The researchers and research assistants (the psychologists) held various joint sessions throughout the study. The major themes emerged from the analysis/interpretation of the data set; they were defined and refined over the period of analysis. Once data were coded and organized thematically, the researchers drew on Howard’s descriptive theoretical decision analysis model [18] in order to favour a better organization and interpretation of the ideas, and also to further understand how the interests, values, preferences, and goals, expressed by the principal actors in the relation oncologist-parent/adolescent, were converted into an effective reference in decision-making, when curative treatment no longer offered any benefit to the adolescent. Three levels of thematic codes were developed: a) a priori themes taken from the guide for interviews; b) emerging themes that emerged in the interviews; and c) analytical themes that grew from the all of the themes. The information generated by means of the interviews was captured for its analysis and managed by use of the computer program Atlas/ti version 7.5.17 (Cincom Systems, Inc., GmbH, Berlin). Inductive and deductive focuses were used to organize and analyse the information contained in each of the narratives.

## Results

Table 1 shows characteristics of the three groups in the study: paediatric-oncologists, parents, and adolescents. The 13 oncologists interviewed had a median of seven years (range: 1–20 years) of work experience and held primary responsibility for providing medical care for the adolescents included in this study. This group was composed of attending oncologists and heads of paediatric oncology services. Thirteen parents also participated in the study; the age range of their adolescents with cancer was from 13 to 18 years old (median: 14 years). Seven of these adolescents had already died; five died from solid tumours and two from acute leukaemia. The interviews with their parents took place a median of 23 months (range: 7–48 months) after the death of the adolescent. The interviews of the remaining six parents, whose children were still alive at the time of the study, took place a median of 280 days (range: 150–365 days) after the attending oncologists told them about the futility of continuing with curative treatment. The six adolescents (median age 15 years; range 13–18 years), who were interviewed, had different types of cancer: solid tumours (*n* = 3), acute leukaemias (*n* = 2), and central nervous system tumour (*n* = 1). Tables 1 and 2.

**Table 1:** Characteristics of all participants involved in the study

Characteristics	Adolescents interviewed (*n* = 6)	Children of parents interviewed (*n* = 13)	Parents ^a^(*n* = 13)	Oncologists (*n* = 13)
	*n*	*n*	*n*	*n*
Age in years, median (range)	15 (13–18)	14 (13–18)	40 (21–60)	38 (32–52)
Males	4	11	3	5
Education				
Uneducated	1	1	0	0
≤Secondary	4	9	5	0
Preparatory	1	3	5	0
Bachelor’s	0	0	2	0
M.D.’s	0	0	0	13
Master’s	0	0	1	7
Diploma course^b^	0	0	0	5
Type of cancer				
Haematological neoplasm^c^	2	2		
Extracranial solid tumour^d^	3	9		
Tumour of the CNS^e^	1	2		
Seven deceased adolescents				
Cause of death				
Treatment-associated complications		1		
Cancer progression		6		
Place of death				
Hospital		3		
Home		4		
Time between disclosure of therapeutic futility and death in days; median (range)			75 (3–365)	
Time between start of non-curative treatment and death in days; median (range)			30 (3–270)	
Duration of interview in min.; median (range)	28 (11–69)		44 (19–134)	51 (17–76)

^a^Parents or primary carers: 8 mothers, 3 fathers, 1 sister, and 1 grandmother

^b^Diploma course in bioethics (*n* = 2); thanatology (*n* = 2); palliative care (*n* = 1)

^c^Acute lymphoblastic leukaemia (*n* = 3); acute myeloid leukaemia (*n* = 1)

^d^Primitive neuroectoderm tumour (*n* = 3); Ewing sarcoma (*n* = 2); osteosarcoma (*n* = 4); testicular germ cell tumour (*n* = 1); colon adenocarcinoma (*n* = 2)

^e^Central nervous system: glioblastoma multiforme (*n* = 1); astrocytoma (*n* = 2)

**Table 2:** Characteristics of the adolescents interviewed

Patient	Type of cancer	Informed on therapeutic futility	Informant	Role adopted in the d-m-p^a^
P32	Hepatic primitive neuroectodermal tumour	No	None	Passive
P33	Colorectal adenocarcinoma	No	None	Passive
P34	Pilocytic astrocytoma	No	None	Passive
P35	Osteosarcoma	Yes	Oncologist	Active
P36	Acute lymphoblastic leukaemia	No	None	Passive
P37	Acute lymphoblastic leukaemia	Yes	Mother	Active

^a^ *d-m-p* decision-making process. Gender: 4 men and 2 women. Age: 13–18 years

The synthesis of the interviews of the 32 participants revealed four themes: 1) the flow of information to inform decision-making; 2) the disclosure of the prognosis; 3) the decision-maker and the stakeholders involved in decision-making (their values, preferences, and beliefs); and 4) barriers and facilitators to decision-making. Some numerical data are presented only to provide the reader a better perspective on the data.

### Information flow (type and amount of information exchanged between oncologist-parents/adolescent)

All the oncologists mentioned that the pertinence of the information is determined in function of the importance that the parents would attribute to it at the moment of deciding whether, in light of the futility of curative treatment, their child should continue, or not, with curative treatment. All the oncologists thought that the announcement of the therapeutic futility places the parents in a psychological state of vulnerability that reduces their capacity to understand the fundamental risk of deciding. Because of this, they said that they preferred that the parents be the ones to determine the type and amount of information that they needed; however, the form in which the information is presented to the parents should be oriented toward what they, as oncologists, considered best for the patient.

*"…The decision is solely medical. The only thing that we can do for the parents is to explain to them why their child will no longer benefit from curative chemotherapies; what palliative chemotherapies are; and why we manage comfort and palliation. That is the information that the parents receive…."* [P19:020102DrGP.doc-29:30]

*"…I think that we limit ourselves, because they enter into shock upon hearing the news. Generally they capture very little, and what they are going to recall of what you tell them is minimal…I do not give them too much information, because I know that they are not going to retain it, and they are not going to understand…."* [P1:010101DrMSAZ.doc-77:77]

*"What is legally required. Talking with them in order to clear up their doubts."* [P4:040101DrAJ.doc-21:21]

*"I speak with the parents and, when they agree upon the decision that they want, they then tell me."* [P3:030101DrAJGQ.doc-166:166]

All the parents spoke of having used the information provided by the oncologists in the decision-making process and of having accepted the recommendations of the oncologists without finding out thoroughly the risks and the benefits.

*"…The oncologist spoke to me and told me that, no, they would not operate on my son, but that they were going to give him six cycles of chemotherapy and, after that, they were going to give him only radiotherapy; then, I said, 'OK.' That is, 'Well, yes'. No? That is, well, now there was nothing left for me but to say, well, 'Yes, it's OK'."* [P8:030101PGQ.doc-50:50]

Of the 13 parents interviewed, six indicated that confidence in the hospital in which their children were being treated was a pivotal element in not having doubts about the treatment given to their children. [P6:010101PGQ.doc-14:14; P7:020101PGQ.doc-126:126; P9:050101PGQ.doc-139:139; P10:040101PGQ.doc-426:426; P23:030103PGP.doc-52:52].

*"I know that this is one of the best hospitals…Therefore, how can I think that there could be malpractice…"* [P8:030101PGQ.doc-114:114]

Two parents stressed that the medical discourse that the oncologist used in communicating the therapeutic futility to them made the information provided incomprehensible. [P7:020101PGQ.doc-56:56; P8:030101PGQ.doc-48:48].

*"…the oncologist told me, 'Very, very difficult times are coming'. But I said to myself…How will I tell you? Well, that it was going to be a tough battle, that is, that it was going to last years…that it was not going to have an end…"* [P7:020101PGQ.doc-56:56]

Two of the six adolescents interviewed knew their poor prognosis. One of them spoke of having been informed of his/her diagnosis, course of treatment and prognosis by his/her mother—at the insistence of his/her attending oncologist [P37:020101AGP.doc-118:124]. Table 2. Another said that he overheard his/her attending oncologist and his/her uncle when they were speaking about the diagnosis, course of treatment and prognosis of his/her disease, at the oncologist’s office. He/she also mentioned having sought information about it on the internet [P35:040301AGP.doc-236:240]. Table 2. The interviews with the oncologists revealed that they inform adolescent only when the parents authorize it; hence they inform the parents first. They act in this way (they said) in adherence to the norms and laws of Mexico. Nonetheless, they (the oncologists) think that it is the adolescent who should make choices about further treatment.

*"It is like a guideline that we have. To inform the parents first, as the ones responsible for their child; thereafter, if the parents authorize it, we inform them (adolescents)…That is our usual procedure…due to the fact that they are minors*." [P13:030102DrGQ.doc-8:8]

*"…We cannot speak directly (to the adolescents), because it is a medico-legal situation, only for that reason…With an incurable disease, the important decision is that of the adolescent…Here, it is not the decision of the parents."* [P14:040102DrGQ.DOC-12:12]

Most of the parents deliberately decided not to inform their children that they were in the terminal phase, whether because they considered them to be too young or because they did not wish to cause their child additional pain or anguish. Of the adolescents interviewed, four [P32, P33, P34, P36] adopted a passive role during their end-of-life period. Table 2. This could have been due to the advanced state of the cancer or to the discomfort resulting from the treatments. They preferred (they said) to hear the information from their parents. Some parents said that their children (13, 14, and 16 years old) were informed at the insistence of the attending oncologist. [P7:020101PGQ.doc-84:84; P10:040101PGQ.doc-136-136; P26:040103PGP.doc-324:338] The parents did not regret having excluded their children from decision-making.

### Disclosure of prognosis

In all cases, the prognosis was presented or explained, to the parents by the oncologists, in terms of the lack of response to curative treatment and of death. The prognosis presentations were oriented toward an explanation of the biological problem and the progression of the disease. The parents faced two possible options for treatment: doing something or doing nothing. The first was a choice of withdrawing, or withholding, curative treatment and providing end-of-life palliative management with or without palliative chemotherapy. The second included the choice of voluntary discharge from the hospital.

Two parents [P7, P8] mentioned that the curative treatment was continued (at the suggestion of the attending oncologist) despite the oncologist’s having communicated that the cancer was in an advanced state. The parents said that it was not until the moment in which the adolescents presented another relapse or cancer progression that the oncologist proposed the withdrawal of the curative treatment and the start of supportive care, because of the low or null probability of cure and the grave condition of the adolescents.

All the oncologists declared that palliative care should be proposed to the parents when they (the oncologists) determined the failure of, or lack of response to, curative treatment. This determination was based on the estimated theoretical-quantitative probabilities of cure (number of lines of treatment employed, number of relapses, and radiological and laboratory evidence).

*"…When the patient has relapsed and I estimate ≤10% probability of a cure."* [P13:030102DrGQ.doc–56:60]

*"…When the patient has not responded to the third or the last line of treatment."* [P19:020102DrGP.doc-19:19; P22:020103DrGP.doc–68:68]

For adolescents with cancer, when treatment is no longer curative, the oncologists diagnosed them as being in terminal stage. It is when, they said, they introduce the idea of palliative care.

*"We broach the subject only at the end, because we are not trained. My preparation is directed more to the prolongation of life and not toward discussing death…."* [P13:030102DrGQ.doc–56:60]

*"A father may say to me, 'You know what? I no longer want the chemo.' He signs the release (discharge from hospital) and takes his sick child. But, that the father should decide who will be moved to palliative care, no. This is by medical consensus."* [P22:020103DRDO:104]

Palliative care—they acknowledged—"…are those medical interventions that do not attempt to cure, but rather try to alleviate the discomfort, pain, and suffering". Thus, when there was no longer a reasonable hope of cure, the oncologists identified as their primary goals: (a) doing no harm by avoiding the adverse effects of curative treatment and (b) the improvement of living conditions of the patient by providing psychological support, reducing the time in hospital, and controlling diverse symptoms by means of palliative chemotherapies, transfusions of blood derivatives, antibiotic therapy, and pain management. All the oncologists, except one [P5:050102DrGQ.doc-26:26], admitted to not having heard the term, therapeutic futility before; so they did not know what it signified. Nevertheless, according to all the oncologists, their judgments about futile treatments emerged from personal clinical experience and experience shared with colleagues and from the cancer treatment protocols existing in their hospitals.

### Decision-makers (their values, preferences and beliefs)

In the relationship oncologist−parents/adolescent, the oncologists interviewed thought that the decision about futility is strictly medical; therefore, they coincided in saying that their role is one of “orienting” the choice of the parents toward what they, as oncologists, consider beneficial for the patient.

*"…when one decides to move the patient to palliative treatment, these are medical decisions; but once the patient is in palliative treatment and the parents or the patient decides not to continue palliative chemotherapy, their decision is respected."* [P19:020102DrGP.doc-37:37]

For all the oncologists, what is important, or valuable, is to cure the disease from the biological point of view or to prolong the life of the patient. Lack of response to curative treatment is considered, by them, as a failure; however, two oncologists said that "…*it is not the doctor who decides if the patient is going to be cured or not, but the disease process*…" [P17:010103DrGP.doc-259:259; P22:020103DrGP.doc-72:72].

Most the parents pointed out that, independently of the type of tumour and the age of their children, they wanted the healthcare professionals, particularly the oncologists and the nurses, who were responsible for the treatment of their children to display an interest in the patient, to explain the situation clearly, and to speak the truth. Similarly, they expressed the need for messages of hope, messages that “*lift the spirits*”. The adolescents interviewed focused on the need for their oncologists to speak to them truthfully.

All the oncologists said that the parents are the ones legally responsible: "*The law is very clear, one cannot override the decisions of the parents*" [P21:040102DrGP.doc-53:53]; nonetheless, they said that they think that the adolescents should be made aware of their impending death. The majority mentioned that it was difficult to specify an age at which the child or adolescent should be informed the poor prognosis; however, there were two exceptions: one oncologist thought that the threshold could be seven years of age; the other, 12 years of age. [P18:010102DrGP.doc-32:35; P22:020103DrGP.doc-142:148].

All the parents agreed that they were the ones legally responsible for their children and that the oncologists are the true decision-makers in such circumstances. The parents spoke of having accepted the palliative management proposed, by the oncologist, when they (the parents) were confronted with the medical information concerning the loss of hope for a cure and with the medical insistence of "*what was finally their (the parents') decision*".

*"…there is really nothing more but to authorize; in the tough decisions, (our participation) is in the authorization…."* [P28:060103PGP.doc-190:190]

*"…they (the oncologists) make the decision, because they are the ones who know how grave the problem is."* [P31:080103PGP.doc-445:447]

Many parents said that they preferred the home as the place for end-of-life care; they stressed their wish to avoid greater suffering and pain by their children. Others preferred that their children remain hospitalized (a) for fear, they said, of facing the symptoms at the moment of their child’s death [P8:030101PGQ.doc-54:54; P27:050103PGP.doc-202:204]; or (b) because they did not understand fully that, medically, their child was now considered to be in the terminal stage [P28:060103PGP.doc-340]. In general, the parents said that the decisions were made within the context of their familial relations and obligations; in some cases, the insistence and preference of the adolescent also influenced the decision [P8, P9, 27, 30]. All the parents and adolescents had strong religious beliefs.

What was important for the adolescents interviewed, who had not been informed about their imminent death [P32, P33, P34, P36], was being cured and continuing to live. When these adolescents stated that they no longer wanted to undergo more chemotherapy (palliative), they were encouraged by their parents to continue the treatment. In contrast, for the two adolescents [P35 and P37] who had been informed of their impending death, what was important was to suffer no longer.

### Barriers and facilitators

The barriers to decision-making most frequently identified by the oncologists were: parental lack of understanding and their difficulty accepting the prognosis, an emotional tie to the patient, and their own lack of training in psychology and/or palliative care. Similarly, they identified the ability to count on a palliative care clinic or service and the availability of an adequate place (not a doctor’s office) to inform the poor prognosis of the disease as being urgently needed. Only two of the parents mentioned as a barrier to decision-making their "*not acknowledging the situation, of not wanting to see…*" [P7:020101PGQ.doc-120:120; P8:030101PGQ.doc-28:28] The two adolescents [P35, P37] who had been informed of their poor prognosis and course of treatment did not mention any barrier in the decision-making.

The facilitators most frequently identified by the oncologists were the progress of the disease and that the father or mother made a firm decision (concerning not to continue curative treatment). For the parents, the facilitators were the prognosis given to them in terms of death, and not wanting to see their child suffer more or undergo a lot of pain. For one of the two adolescents [P35, P37], the facilitators included to have heard of the prognosis in terms of probabilities of death in the short term and to have previously obtained information about the disease from the Internet [P35:040301AGP.doc:48:50] and, for the other, to learn of the prognosis in terms of null possibility of cure [P37:020101AGP.doc-118:124].

## Discussion

The findings of this study suggest that, in the relationship oncologist-parents/adolescent, the oncologists recognized that it was their duty to provide the parents/adolescents with the information that they (the oncologists) considered to be appropriate and relevant, thereby permitting the parent/adolescent to have “control” over the course of action that was to be followed; that is, the oncologists believed that their role is to orient the choice, making recommendation(s), and to give the parents/adolescents the opportunity to decide whether they accept, or not, the recommendation(s). On the one hand, this reflects a certain model of paternalism, because healthcare provision is tailored to the preferences of the attending oncologists. On the other hand, this reflects the oncologists’ belief that autonomous choice is a parental right that should be acknowledged and their personal choices respected. Beauchamp and Childress [22], Seedhouse [23], Lain [24], among others, have stated that autonomy is not a single right that can be ceded; rather, it is an intrinsic personal quality that can be enhanced, or diminished, depending upon what happens to or is done to people.

Our findings also indicate that the oncologists believe that the parents would have difficulty in making an appropriate, reasoned choice after the disclosure of therapeutic futility. This claim is based on unreliable personal criteria developed during a somewhat uncritical personal experience; for example, the oncologists said that they limited the amount of information presented, because they thought that "what the parents would remember will be minimal". In addition, they considered that the decision-making capacity of the parents was compromised and, also, that parents had little capacity to understand the scientific objectives and procedures of medical treatment. Therefore, they prefer to disclose the information about prognosis in negative terms (e.g., "your child no longer will benefit from curative chemotherapy") to induce a parental response that will allow them (the oncologists) to act paternalistically by protecting parents and, in the end, the adolescents, against potentially harming consequences of their own (parents and adolescents) decisions (i.e., therapeutic obstination). In this respect, Beauchamp and Childress [22] and Seedhouse [23], among others, have pointed out that the dialogue with parents/adolescent is not about communicating all the potentially relevant information, but rather guaranteeing a realistic comprehension of the important information. This implies helping the parents (in the present context) to reach a point at which they can make a reasoned and responsible choice; otherwise, it makes no sense to speak about respect for autonomy as an ethics principle when what is meant is the legal or institutionally valid authorization by the parents.

The fact that all the parents accepted the recommendations or the plan proposed by the attending oncologist *(*i.e., to suspend curative treatment and to start palliative management of the disease, without enquiring more into the risks and benefits) did not necessarily signify that that was what the parents really wanted. The respectful acceptance of medical recommendations is part of the value of courtesy and deference to authority that is characteristic of the Mexican culture [5, 25]. Directly contradicting a physician is considered to be a disrespectful and discourteous [5]. Klessing [5], Schuler [25], Napoles [26], Gao [27], among others, report that the extent to which one accepts unequal power relations, or ‘power distance’, denotes cultural configuration of the role expectation that govern social and interpersonal relationships. Thereby, it would be expected that patients from cultural groups characterized by ‘high power-distance’, like those in Mexico and Latin America countries, accept authoritative and “expert” recommendations from their doctors. Different from low power-distance culture, like the U.S., in which a patient from this type of cultural background would expect to share opinions, concerns, and beliefs with their doctor. Nonetheless, in the face of cases of disagreement between oncologist-parents/adolescent (e.g., when the parents insist in continuing curative treatment, or when the parents refuse to take the child from the hospital for fear that their child would die at home) the oncologists prefer to avoid possible legal repercussions by giving greater value to the obligation to respect the desires of the parents than to the medical value of beneficence. This behaviour manifests what is legally required —respect for the autonomy of parents/carers even if the oncologist disagrees with their views or actions [15]. However, ethics calls for oncologists to go beyond what is legally required and, as it has been mentioned, help the parents to rich a point at which they can make reasoned and responsible choice [22, 23].

Despite current guidelines in paediatric palliative care advocacy in the adolescent with capacity to make decisions about her/his medical treatment, including treatment during the terminal stage of cancer [15, 16, 28], the current study showed that the parents interviewed did not consider it necessary to inform the adolescents about their impending death. This appears to be congruent with the fact that parents were terrified after being informed that curative treatment for the cancer was no longer an option and then seeing their child in pain and with other unpleasant symptoms. In these circumstances the last thing that parents wanted was to make their child aware that curative treatment had failed. Also, to be considered is the fact that Mexicans have a familial orientation and the person always has to be thought of as a member of a family. This assertion is supported by other studies [29, 30] that report on the importance of family in decision-making at the end-of-life in Latin-American families and on the belief that truth-telling about prognosis is harmful to the patient; hence, Latin-American families, and Mexican families by inclusion, prefer to not discuss death openly. Understanding this cultural preference is important, because it forestalls us, and rightfully so, from the risk of assuming that respect for autonomy is an absolute moral obligation, as discussed below, and also from the risk of placing too much weight on top-down guidelines [15, 16] as the source of moral authority.

This study also revealed that the oncologists prescribed the suspension of curative treatment, and the start of palliative management of the cancer, when they concluded (based on tangible facts such as the number of lines of treatment employed, number of relapses, and radiological and laboratory evidence) that curative treatment was no longer beneficial. The consequent ethical question asks if, in face of therapeutic futility, the biological progression of the disease is the only important concern. This question raises some points that deserve discussion.

First, therapeutic futility is the expression of the combination of a scientific judgment and a value judgment (by the oncologist) in the sense that it acknowledges the uniqueness of the individual (the course of the disease, values, context and the physiological idiosyncrasies) [22]. The ethical and practical problem occurs when only the oncologist’s own values and objectives are considered when making the judgments. The parents’/adolescents’ perceptions of harm and benefit are idiosyncratic, peculiar to each patient or family, and depend on their way of life, on how they perceive themselves, and on the emotions and the perceptions awaken in them. Therefore, consideration of the needs, values, and preferences of the parents/adolescents is part of the medical principle of beneficence—a principle professed by the oncologists. Lain [24] and Campbell [31], among others, point out that, in a doctor-patient/family relationship, a co-operative relationship (co-execution) between the parties is indispensible.

Second, the foregoing leads naturally to another argument. Within the context of therapeutic futility, when beneficence is technically conceived and realized appropriately, it takes precedence over respect of autonomy of parents/adolescents. Beneficence is not just to do with physical functioning; it is at least equally to do with the mental life of a person/patient [22, 23]. The last statement is important in making decisions when treatment is futile. For instance, it would be inappropriate if a parent, in the face of therapeutic futility, were to insist that curative treatment be continued. In such case, the principle of beneficence would supersede the principle of respect for autonomy. Even though the parents are the primary decision makers and the oncologists have the obligation to respect parental choices, when parental preferences increase the risk that their adolescent children will be harmed, such preferences are inappropriate. This should not be read simply as attempting to maximize physical benefit or minimize suffering. To educate parents/carers about the effect of treatment on their child body, and ensuring that an extremely stressed parent/carer does not become a victim of carer burden by allowing her/him to take a rest, are both actions within a continuum that would qualify as a process to enable autonomous parents/carers. Education for health is not just to do with physical functioning, it is work for reaching wholeness; that is why ethics is process-based and dialogical, rather that rule-based.

Third, although all the oncologists considered it important “not to cause more harm” to the adolescent with terminal stage cancer, the fact that some oncologists preferred to prescribe further curative chemotherapy seemed to depend more on the importance they attached to either curing the cancer or prolonging a patient’s life. These findings are consistent with the report of Buiting et al. [32] that shows that the tendency of oncologists to extend curative chemotherapy until the terminal stage of cancer, and to strive to prolong the patients’ life, could be explained not only by patients’ and oncologists’ mutually reinforcing attitudes of not abandoning curative treatment; but also, by the oncologists’ belief that removing a patient’s hope by withdrawing or withholding curative treatment is harmful. It should be mentioned that these findings do not solve the controversy concerning quantity versus quality of life. Nonetheless, the fact that the judgments concerning the correct course of action, in the face of therapeutic futility, always should be specific to each individual patient does not mean that these judgments are simple matters of opinion or solely to be determined by the personal desires of the parents and/or adolescents. A judgment of futility requires that the diagnosis and prognosis be based on the best available research evidence along with a continuous and forthright dialogue between the oncologist and the parents/adolescent. The dialogue must lead the oncologist to understand the entire personal and social reality of the patient and his/her family and must also allow the parents and the adolescents to understand what is happening and what is possible and what is not possible. The preferences of the parents/adolescents are crucially important, but not necessarily decisive.

According to the participating oncologists, the palliative management of cancer should begin when adolescents become terminally ill; i.e., when due to the biological progression of the disease, they determine that the treatments have little or null probability of success and a higher probability of being more prejudicial that beneficial. Inherent assumption to the biomedical model, which is based on an objective science, in that explanations of disease focus on biological changes to the relative neglect of social and psychological factors [33].

Most oncologists identified the following as their major barriers for the determination of, and timely communication about, the terminal cancer stage: their own poor training in conducting end-of-life conversations; the emotional ties with adolescents they had known since childhood; the problem of having to confront some parents who “do not understand” and “do not accept the prognosis”; and the uncertainty in prognostication per se. These results are consistent with those reported in other studies [34, 35], which highlight several factors associated with the attitudes of the physicians that influence the determination of the moment to communicate that the adolescent is at the terminal cancer stage. Among such factors are: the difficulty in arriving at an accurate and precise prognosis, poor or lack of training in communication concerning end-of-life issues and discussions about them, discomfort about talking about poor prognosis, and about death and dying because it provokes emotions difficult to manage by all participants, and the time available for such communication.

The oncologists recognized that palliative care is of great significance, because it can improve the life conditions of adolescents with incurable cancer. That is why they identified the following as urgent needs for their hospitals: having a clinic or palliative care service for referral of patients, an appropriate place to discuss therapeutic futility, and training (for them, the oncologists) in psychology and/or palliative care. As to the idea that the optimal place for the terminal stage is at home, the current policy of the Mexican government is that patients should be supported to die at home, should that be their preference [15]. All the participating oncologists attributed immense importance to the home as final place for end-of-life care. But if such importance is given to the home, then appropriate support mechanisms are required for adolescents and their parents/primary carers, such as educating families on what to expect; connecting families to healthcare professionals and community support networks; bolstering parent coping and developing parental problem-solving skills; as well as psychosocial screenings to identify family and patient risk factors to support the needs of patients [36]. However, some of the participating parents preferred the hospital, either because they feared that at home they would be providing little control of the symptoms or because they placed their fears and anxieties concerning the control of pain and symptomology at the last moments of their child’s life ahead of anything else. Guidelines for palliative care [15] are necessary, because they establish what should be an appropriate professional conduct; however, they are not enough. Training in ethics must be provided to assist the oncologists in achieving and maintaining ethical and technical professional standards.

It is relevant to mention that there was a difference among the values of the oncologists, the parents, and the adolescents. Oncologists valued curing the disease and prolonging the patients’ life. They considered it important that the adolescents be involved in the decision-making. In contrast, the parents valued honesty in communication, clarity of the information provided to them, and an oncologist with a deep involvement in their relationships; for them, respect for the autonomy of their child was not as important. Likewise, the adolescents said that they valued honesty from their oncologists. The qualities valued and demanded by the parents and adolescents pertain to the principles of beneficence and non-maleficence and serve as a framework for understanding what the participants tended to value at end-of-life. Awareness of this diversity of values at play can help to avoid important clinical consequences in the oncologist−parents/adolescent relationship, such as: difficulties with informed consent, resentment toward a detached doctor, decreased satisfaction with care, and miscommunication.

### Limitations

All participants were recruited from paediatric oncology services of three tertiary-care medical centres located in Mexico City. The participating hospitals belong to the public healthcare sector. The public sector provides healthcare to ~95% of Mexicans, with the private sector serving the remaining population [10]. Thus, the results of this study may not be extrapolated to adolescents attended in the private sector.

This study relies solely on semi-structured, in-depth interviews data from the main agents of the decision-making process. This could be seen as a limitation to the full understanding of the *emic* perspective on the Mexican culture—as we did not include more ethnographic techniques for data generation or multiple sources of data. Nonetheless, the fact that (a) the participating oncologists were of different genders, ages, and work experience; (b) the participating parents/carers and adolescents were of different genders, ages, educational background; (c) the adolescents had distinct types of tumours; and (d) the participating hospitals are national referral medical centres that provide medical care to patients from various parts of Mexico, provide a good foundation for developing a better understanding of how the decision-making process on therapeutic futility is carried out in Mexican adolescents with cancer. It is also important to note that this study is not generalizable in the same sense of quantitative research, because it involves non-random, purposive sample of individuals who contributed to the generation of data. Additional research is required to establish whether these findings are indeed generalizable to other settings.

It should be mentioned that extreme care was taken in the methodological rigour with which this research was performed in order to reduce potential biases that are characteristic of semi-structured interviews. The methods used and the active focus of the process of research that was carried out guaranteed the representativeness of the sample. In brief, the active focus of the research refers to the identification of the question that forced the researchers to think; to confirm or to refute; to gather more data; and to pursue emerging paths of research [19].

More qualitative and quantitative empirical work is needed to explore the advantages, disadvantages and the inconveniences of adhering, or not, to the recommendations of the policy-makers about involving adolescents with incurable cancer in decision-making concerning their treatment, or at least, such work is needed to identify the factors that favour the participation of adolescents in this decision-making process. Decisions are the result of an act of willing by a human being [18]. A human being (here, oncologists, parents, and adolescents) need data, and also also needs to understand the data and to judge whether or not the understanding is correct to make a judgment value and a decision to choose and bring about what is valued; therefore, an oncologist cannot limit herself or himself to objectifying and specifying what he or she finds in the patient. A patient is not a collection of body parts or a whole with idiosyncratic desires. Hence, more empirical work must be carried out to find out how contextual and semantic factors influence parents’ and adolescents’ perceptions of the information received from oncologists and how the key aspects of decision-making that remain unexplored, such as the interaction between individual and organizational ethical values, shape the nature of decision-making.

## Conclusion

The knowledge generated in this study is valuable, because it permits to understand how the decision-making process is understood and constructed by the principal agents in a terminal cancer, especially when the decisions have great consequences not only for the sick adolescent, but also for the family. Also, it provides evidence of the compelling and urgent need for undertaking a more effective implementation of palliative care, especially in developing countries such as Mexico. Likewise, it shows that appropriate training and continual education in the ethics of quotidian clinical practice is indispensible in order to achieve a good medical practice.

## Additional file

Additional file 1: Topic guide. Outline of the topic guide. An interview topic guide. (DOCX 34 kb)
