# Supplementary material for: Decision-making on therapeutic futility in Mexican adolescents with cancer: a qualitative study
Source: BMC Med Ethics. 2017 Dec 11;18:74. doi: 10.1186/s12910-017-0231-8 (PMC5724238; doi:10.1186/s12910-017-0231-8)
Supplement: Additional file 1: — Topic guide. Outline of the topic guide. An interview topic guide. (DOCX 34 kb) [file 12910_2017_231_MOESM1_ESM.docx]

**Additional file 1: Outline of the topic guide**

The following is a topic guide used to prompt participants to openly convey their viewpoints regarding the decision-making process when cancer treatment is no longer curative. It was not intended that each of the following questions be asked word by word, but rather that they be used as a memory aid to ensure that all important areas would be covered.

**Start interview for all participants**

Introduction of the interviewer, the study, and its purpose

Estimated duration of the interview

Consent and confidentiality: written consent, audio recording, how the data will be used

Possibility to stop at any time, or decline to answer any questions without any consequences

**Background for all participants**

Demographic data

**Interviews with paediatric-oncologists**

For all participating oncologists, the interview started with the question: can you tell me about your clinical experience with patients with cancer when curative treatment has been exhausted? Responses were usually lengthy. When necessary, prompts were used to cover the following points:

- When do you realize (your patient) is not going to get any better? How do you come to realize this?
- How do you carry out the disclosure of prognosis? Do you find that the amount of information you present is just the right amount of information?
- Whom do you usually inform when you find that curative treatment has been exhausted?
- Whom do you usually involve in the treatment decision, when curative treatment has been exhausted?
- Do you involve your patients in the decision-making process?
- What aspects of context, attitudes, and experience facilitate or constrain decision-making?
- What do you understand by the term ‘palliative care’? And, by the term ‘terminal care’?

**Interviews with parents or carers**

For all participating parents or carers, the interview started with the question: I would like you to tell me about the medical condition of your child. What do you know about it? Responses were usually lengthy. When necessary, prompts were used to cover the following points:

- How did you learn about the current prognosis (of your child)?
- Did you find that the amount of received information was just the right amount of information?
- How much did all the received information improve your understanding of the prognosis and of treatment options that might be available?
- How much did your child know about her/his disease and prognosis?
- As for withdrawal of cancer curative treatment, how was the final decision made? Who participated? How much did your child participate?
- What was most important to you, your child, and your family during the last few months of your child’s life? Or, now (in case the child is still alive) that you know that your child is terminally ill?
- What aspects of the healthcare provided were (or, have been, in case the child is still alive) most helpful to you, your child, and your family?
- Are you satisfied with the decision made?
- Where did your child die? Was this in line with your (and your child's) wishes? Or, where would you like your child to die?
- Did you speak (or have you spoken, in case the child is still alive) to your child about their own death?

**Interviews with adolescents**

For all participating adolescents, the interview started with the question: I would like you to tell me how your disease is going? What do you know about it? Responses were usually lengthy. When necessary, prompts were used to cover the following points:

- How did you learn about what is happening?
- Did you find that the amount of received information was just the right amount of information?
- How much did all the information improve your understanding of your medical condition?
- How much did all the information improve your understanding of the treatment options that might be available?
- Do you like to be informed about your disease? Do you think this is important for you?
- Who are the decision-makers about your treatment? How are decisions made?
- Are you satisfied / comfortable with the decisions made?
- How do you see yourself in the near future?
